# Females in Psychedelic Research: A Perspective for Advancing Research and Practice

**DOI:** 10.1021/acsptsci.5c00255

**Published:** 2025-06-05

**Authors:** Zahira Ziva Cohen, Grace Blest-Hopley

**Affiliations:** † Medical Research Council − Cognition and Brain Sciences Unit, 2152 University of Cambridge, CB2 7EF Cambridge, U.K.; ‡ Department of Brain Sciences, Faculty of Medicine, Imperial College London, W12 0NN London, U.K.; § Department of Psychosis Studies, Institute of Psychiatry, Psychology & Neuroscience, 5000 King’s College London, SE5 8AB London, U.K.

**Keywords:** psychedelics, psilocybin, menstrual Cycle, progesterone, estrogen

## Abstract

The influence of ovarian hormone fluctuations on neurochemistry, cognition, and psychological responses remains insufficiently examined in current psychedelic research and clinical protocols. Traditional practices and case studies underscore the importance of accounting for these factors in investigations of psychedelic effects. This opinion paper explores the critical intersections between female hormones and psychedelic experiences, informing improved research and practice. Estradiol (E2) and progesterone (P4), the primary ovarian hormones, modulate neurotransmitter systems central to psychedelic pharmacology, including serotonin (5-HT), dopamine, GABA, and glutamate. These hormonal interactions affect interhemispheric communication, synaptic plasticity, mood, cognition, and behavior. Fluctuations across the menstrual cycle influence 5-HT2A receptor expression and functional connectivity, potentially modulating both the subjective intensity and therapeutic efficacy of psychedelics. Additionally, oscillations in female hormones across the menstrual and life cycles affect mindset, a significant factor in safe and effective psychedelic use. These findings suggest that female hormonal variability may play a pivotal role in psychedelic experiences. Incorporating menstrual phase tracking and hormonal assays in both clinical trials and observational studies can reduce data variability, support individualized care, and improve informed consent practices. This would improve data integrity and ensure that women are fully informed about the potential influence of their hormonal state on their psychedelic experience, supporting truly informed consent. This paper emphasizes the need for an improved understanding of the complex interplay between female-specific biology and psychedelic pharmacodynamics to advance safe, ethical, and effective psychedelic research and therapies for women.

## Graphical abstract

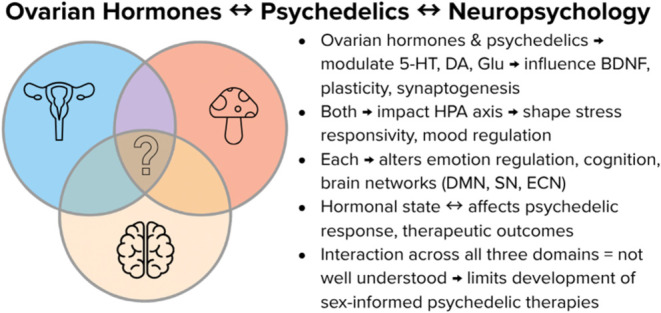

The evolution of human beings has optimized the survival of the species and with it, differences in biology exist between the sexes. Furthermore, differences between the sexes go beyond reproduction and emerge in the neuro-psycho-socio-cultural fields.^1–4^ One central and relevant interactive system is the reproductive system, hormonal cyclic changes during the menstrual cycle (MC), and other physiological systems. Nevertheless, these interactions have not been adequately studied or understood. The measurable day-to-day hormonal fluctuations need to be addressed in relation to their potential impact on treatment outcomes and participant responses in psychedelic research.

In the re-emerging field of psychedelic science, there is a need for consideration of hormones and the MC, as has been done in longstanding Indigenous practices with psychedelics.^5^ A recent case study suggests a possible interaction between MC and psychedelic use,^6^ which is supported by earlier preclinical studies.^7^ Psychedelics’ therapeutic action increasingly shows the importance of mood or mindset^8^ and hormone interaction.^9^ However, women’s characteristics are not currently being appropriately considered.

This opinion paper aims to set out theoretical and experiential knowledge about female neuro-psychobiology, providing insight and hypotheses to advance safe, ethical, and effective practices for psychedelic research and therapy.

## 1 Observations

### 1.1 Ovarian Hormones and Neuropsychological Functioning

The impact of ovarian hormones and the MC on mental health and brain functioning has both socio-cultural^2,3^ and biological origins.^1,4^ Although the field is still developing, and many mechanisms remain to be fully understood, current evidence highlights several of the most consistently observed links between menstrual cycle phases and fluctuations in psychiatric and psychological symptoms.

Studies show that the premenstrual and menstrual phases (i.e., perimenstrually) are associated with symptom exacerbation across a range of mental health conditions.^10^ Depression and suicidality intensify premenstrually, with over 60% of women with major depressive disorder experiencing premenstrual symptom exacerbation (PME), leading to longer episodes and more severe symptoms.^10,11^ Anxiety disorders—including panic disorder, post-traumatic stress disorder, social anxiety disorder, and obsessive-compulsive disorder—similarly fluctuate, with increased symptom severity during perimenstrual periods.^10,12^ Specific conditions show distinct patterns: fibromyalgia patients experience heightened pain and negative affect and less positive affect in the luteal phase,^13^ eating disorders display altered stress responses following meal-skipping based on menstrual regularity, and increased binge eating during the luteal phase,^10,14^ and increased alcohol and nicotine use perimenstrually is found mainly in individuals with premenstrual dysphoric disorder (PMDD) and premenstrual syndrome (PMS), suggesting hormonally driven self-medication tendencies.^10,15^

Ovarian hormones are circulated in the body and locally synthesized in the brain. Serving as neuroactive steroids, they can alter brain function and structure directly^16^ or indirectly, through the serotonergic,^17^ dopaminergic,^18^ cholinergic,^19^ noradrenergic,^20^ glutamatergic^21^ and GABAergic^22^ systems.^23^

Estradiol (E2) and progesterone (P4) regulate the serotonin system, including the 5-HT2A receptor, through both genomic and nongenomic mechanisms.^17,24,25^ Genomically, E2 binds to intracellular estrogen receptors—particularly ERβ in serotonin neurons—initiating transcriptional cascades that increase HTR2A (the gene for the 5-HT2A receptor) mRNA levels and upregulate 5-HT2A receptor expression. This is thought to occur via estrogen response elements (EREs) in the HTR2A promoter and through interactions with transcription factors such as AP-1 and Sp1.^25^ These effects have been observed in mood-related regions including the prefrontal cortex and amygdala.^17,24^

P4 can either enhance or attenuate E2-driven receptor expression, depending on timing and hormonal levels—for example, when both hormones are elevated during the luteal phase.^26^ Animal studies show increased receptor density in the olfactory cortex, dorsal raphe, nucleus accumbens, and amygdala following E2 exposure.^17^ Nongenomic modulation occurs via membrane-bound estrogen receptors, post-translational changes, and trans-synaptic effects,^24,25,27^ while P4 metabolites act on GABA-A receptors, representing a separate neuromodulatory mechanism.^17^

Polymorphisms in estrogen receptor genes have been linked to psychiatric vulnerability in women. ERα variants such as PvuII (rs9340799), XbaI (rs223493), and the TA-repeat are associated with neuroticism, anxiety, and increased risk for mood disorders including premenstrual dysphoric disorder and depression.^28,29^ ERβ polymorphisms (e.g., G1082A, A1730G, and short CA repeats) have been linked to anorexia and depression, while the P4 receptor variant G331A has been associated with panic disorder.^28^

E2 also regulates brain-derived neurotrophic factor (BDNF), particularly in the hippocampus. Neuroimaging studies show that E2 interacts with the BDNF Val66Met polymorphism to modulate hippocampal activation during working memory tasks, with altered recruitment seen in female Met carriers.^30^ These findings highlight gene-by-hormone interactions relevant to sex differences in mood disorders.

Enzymatic pathways also shape estrogen signaling in the brain. Aromatase, expressed in regions including the hypothalamus, cortex, and hippocampus, converts androgens to estrogens and produces catechol estrogens via hydroxylation.^1,31–33^ Catechol estrogens act as competitive inhibitors of catechol-O-methyltransferase (COMT), which metabolizes estrogens and is modulated by functional variants such as Val158Met.^34–37^ These interactions can influence cognition, emotional regulation, and estrogen sensitivity.

While individual genetic variability interacts with the serotonergic system to confer either resilience or vulnerability to mood disorders,^28–30^ the effects of ovarian steroids extend beyond receptor-level modulation. At the systems level, ovarian hormones influence brain structure, function, and plasticity by regulating synaptogenesis, pruning, and neurogenesis across multiple regions.^38–40^ E2 and P4 modulate structural plasticity, functional connectivity, and remodeling in areas central to emotion and cognition, including the hippocampus, amygdala, and prefrontal cortex.^39,41,42^ E2 can exert rapid or prolonged effects on neuroplasticity,^43,44^ increasing dendritic spine density, enhancing synaptic strength, and upregulating BDNF.^44–46^ P4 also promotes synaptic remodeling, with elevated levels associated with increased spine density on hippocampal neurons.^39^

Ovarian hormones enhance cortico-cortical and subcortico-cortical connectivity,^47,48^ support myelination, and are linked to increased hippocampal and frontal gray matter.^48,49^ Functional connectivity fluctuates across the menstrual cycle; higher P4 levels correlate with stronger connectivity between the dorsolateral prefrontal cortex and hippocampus.^47,49^ Dynamic changes are also observed in the default mode, executive control, and salience networks across cycle phases.^50,51^ Elevated E2 and P4 levels are further associated with increased interhemispheric communication and reduced lateralization, supporting bilateral engagement during cognitive tasks.^52,53^ These plasticity-promoting effects, while adaptive, may also heighten vulnerability in genetically susceptible individuals. The same mechanisms that support integration and flexibility may contribute to dysregulation when hormonal shifts interact with molecular risk factors.

Starting their impact in the womb, the mother’s E2 and P4 influence the development of the unborn and newborn brain.^54,55^ In the prenatal and early postnatal period, E2 shapes neural circuits by promoting synaptogenesis and preventing programmed cell death, particularly in the hypothalamus and limbic system, during what are considered sensitive periods of brain organization.^55^ The second stage, when sex hormones are imminent, is the transition through puberty to adulthood. Ovarian hormones are critical for the organization of the developing female brain, and influence behavioral and affective changes in this developmental stage.^56,57^ During adolescence, these hormones refine functional connectivity in the prefrontal and temporal cortices through synaptic pruning and circuit remodeling, supporting executive function and emotional regulation.^58^ P4 additionally modulates inhibitory tone by enhancing GABA-A receptor activity via its neurosteroid metabolite allopregnanolone, which contributes to emotional regulation and synaptic balance.^59^ Following the reproductive years, when ovarian hormones have a recurrent predictable effect on female’s neuropsychology, are the perimenopausal and menopausal stages during which ovarian hormone levels decrease, exposing females to neurocognitive and neuroaffective challenges, partly due to reduced synaptic plasticity, altered neurotrophic support, and changes in functional connectivity within prefrontal-limbic circuits.^58^ Together, these stage-specific influences of ovarian hormones highlight the lifelong role of endocrine modulation in shaping female brain development and emotional regulation, establishing critical windows during which hormonal disruption or sensitivity may contribute to psychiatric vulnerability.

Fluctuation of ovarian hormones, E2 and P4, has an impact on psychosocial stress, increasing psychiatric vulnerability in different menstrual phases.^60,61^ The stress system is crucial in diathesis-stress models of psychopathology interacting with hormonal fluctuations.^60,62^ Stress impacts brain and behavior through the hypothalamic-pituitary-adrenal (HPA) axis, causing widespread hormonal, neurochemical, and physiological changes.^63^ The complex interplay between stress and menstrual health is evidenced, for example, in cases of high stress among females with a history of dysmenorrhea^64^ or in the higher percentage of mood disorders in females in the period from menarche to menopause.^65^

The HPA axis plays a central in regulating the secretion of stress-related hormones such as corticotropin-releasing hormone, adrenocorticotropic hormone, and glucocorticoids.^66^ The HPA axis also interacts with the hypothalamo–pituitary–gonadal (HPG) axis, which governs the production of gonadal steroid hormones.^66,67^ Evidence shows that HPA axis function differs between men and women,^67–69^ with that dysregulation of this system is commonly found in stress-related psychiatric disorders,^68–70^ which are more prevalent in women.^68,69,71^ This sex difference in HPA function may reflect hormonal modulation across phases of the menstrual cycle.^67,68^ It is also associated with depressive states,^69^ which can lead to change in HPA activity, such as elevated basal cortisol or altered reactivity to stressors.^68,69^ E2 and P4 influence the HPA axis through distinct mechanisms, and their fluctuations are key contributors to sex-specific vulnerability to stress-related psychiatric vulnerability.^67,71–73^ E2, depending on receptor subtype (ERα vs ERβ) and context, can either enhance or attenuate HPA reactivity.^67^ In contrast, P4 – primarily through its GABAergic metabolite allopregnanolone – typically inhibits HPA activation.^67,74^ These hormonal dynamics translate into clinically relevant differences: periods marked by low E2 or abrupt P4 withdrawal (e.g., the premenstrual or postpartum periods) linked to heightened risks of depression, anxiety, and PTSD symptoms.^70,71^ Such phases also correspond to altered HPA function.^69,70^ Conversely, high E2 states may facilitate fear extinction and may offer some protection against trauma-related psychopathology.^67,70–73^

Taken together, the converging evidence from neurobiological, genetic, and neurodevelopmental studies underscores that ovarian hormones are not ancillary variables but central modulators of brain function, emotional regulation, and psychiatric vulnerability across the lifespan. Their fluctuating nature—especially across the menstrual cycle and hormonal transitions—interacts with psychiatric symptom expression, modulates key neurotransmitter systems, and directly alters brain structure and connectivity. In particular, E2 and P4 critically regulate the serotonergic system, including dynamic control of 5-HT2A receptor expression and function, linking hormonal shifts to affective dysregulation and stress sensitivity. These endocrine effects intersect with individual genetic variation, influencing receptor polymorphisms, neuroplasticity, and large-scale network activity.

### 1.2 Neuro-Psychological Mechanisms of Psychedelics

Psychedelics (often derived from certain plants, animals, and fungi) can be divided into three categories based on their chemical structures: (1) tryptamines, (2) ergolines, and (3) phenethylamines.^75^ Here we focus on classic psychedelics, which primarily act via the 5-HT2A receptor. While some drugs, such as THC,^76^ ketamine,^77^ and MDMA^78^ produce similar altered states and are sometimes called psychedelics, they do not share this primary site of action, so our discussion here is limited to classic psychedelic compounds. Our discussion here is limited to classic psychedelics, which exert their primary effects through agonist (including partial agonist) activity at the 5-HT2A receptor. Our discussion does not include consideration of psychostimulants such as MDMA which act primarily as a serotonin releaser and interact uniquely with neurotransmitter systems,^78^ or dissociatives like ketamine, which act primarily on the GABA and glutaminergic system.^77^ Notably, much of the mechanistic understanding of psychedelic action is derived from preclinical studies conducted exclusively in male animals, limiting the generalizability of our understanding.

Classic psychedelics are agonists at the 5-HT2A serotonergic receptor,^79^ modulating 5-HT2A receptor-linked signaling pathways.^80^ Psychedelics also engage other serotonergic receptor subtypes, including 5-HT1A/B/D/E, 2B, 5, 6, and 7.^81^ The diversity observed among psychedelic effects is thought to result from their unique efficacy at each intracellular signaling cascade. Distinctive binding affinities at these serotonin receptor subtypes further contribute to this variability.^82,83^ Psychedelics interact with 5-HT1A receptors, which are coexpressed with 5-HT2A receptors; notably, these receptors couple to different G proteins that they preferentially signal through. Both 5-HT1A and 5-HT2A receptors are expressed in regions implicated in cognition and memory processing,^79^ as well as in the modulation of mood and anxiety.^84^ 5-HT2A receptors are densely expressed postsynaptically on layer V pyramidal neurons and GABAergic interneurons in the neocortex.^85^

Beyond their primary action on the serotonergic system, classic psychedelics’ binding to 5-HT2A receptors stimulates glutamate release, subsequently activating AMPA receptors and enhancing glutamatergic activity in the cortex.^86,87^ Furthermore, classic psychedelics indirectly modulate the GABAergic system, often increasing cortical GABA and glutamate levels, which may underlie the reported anxiolytic effects of these compounds.^88,89^ This modulation may involve activation of 5-HT2A and 5-HT2C receptors on GABAergic interneurons in the prefrontal cortex,^89^ although the specific mechanisms depend on the receptor profile of each compound.

Classic psychedelics interact with the dopaminergic system, directly through the D1 and D2 receptors,^81^ and indirectly via the trace amine-associated receptor 1 (TAAR1), which exerts an inhibitory effect on dopaminergic activity.^90^ For example, lysergic acid diethylamide (LSD) and psilocybin elevate extracellular dopamine levels in the nucleus accumbens, a region implicated in mood regulation and reward processing.^89,91^ Classic psychedelics have also been shown to influence the oxytocinergic system. For example, LSD significantly increases circulating oxytocin levels via 5-HT2A receptor activation, which has been associated with acute changes in social-emotional processing.^92^ While that study included both male and female participants, no sex-based analyses of oxytocin response were reported. These findings suggest that psychedelic-induced oxytocin release may support their prosocial and empathogenic effects, adding to the complexity of their neuropsychological action.

While secondary to their serotonergic effects, interactions with the GABAergic and dopaminergic systems contribute to the complex psychological and physiological profiles of classic psychedelics. Notably, dimethyltryptamine (DMT) exhibits neuroprotective properties through sigma-1 receptor activation, underscoring the multifaceted receptor activity of these compounds.^93^ At high doses, LSD also engages adrenergic receptors, further adding to its complex pharmacological effects.^94^

Psychedelics also modulate synaptogenesis, neurogenesis, and neuronal plasticity.^79,82,95^ These effects are mediated through upregulation of the proto-oncogene c-fos and brain-derived neurotrophic factor (BDNF), along with activation of tyrosine kinase B (TrkB) receptors and the mammalian target of rapamycin (mTOR) signaling pathway.^96,97^ The dendritic spine-promoting properties of psychedelics appear to be mediated by 5-HT2A receptor activation.

In addition, psychedelics also influence immune signaling by suppressing pro-inflammatory cytokine production and reducing microglial activation, processes that contribute to the maintenance of chronic neuroinflammation.^98^

Psychedelics may influence the hypothalamic-pituitary-adrenal (HPA) axis through mechanisms involving serotonin, dopamine, and sigma-1 receptor signaling,^9,99^ as well as through regulation of corticotropin-releasing factor in the hypothalamus and modulation of brain regions that control HPA activity.^100^

Beyond molecular and cellular effects, psychedelics also act at the systems level to alter cortical network dynamics. Activation of 5-HT2A receptors on layer V pyramidal neurons in the prefrontal cortex increases excitatory signaling and drives heightened activity across higher-order cortical regions.^101^ This leads to transient disruption of large-scale networks—particularly the default mode, salience, and frontoparietal control networks—associated with self-referential thought and cognitive regulation.^101,102^

These network-level changes are thought to reduce the precision of top-down predictions within a hierarchical predictive coding framework, relaxing high-level priors and allowing for unconstrained information flow.^103^ This may mechanistically underlie core psychedelic experiences such as ego dissolution, altered perception, and mystical-type states—linking receptor-level pharmacology to complex changes in consciousness.

The experiential effects of psychedelics are further shaped by psychological and environmental factors, such as expectations, setting, and emotional state, which can amplify or diminish specific neurobiological responses.

Set and setting—referring to the internal mindset and external environment, respectively—are crucial to the psychological mechanisms of psychedelics. The psychological state, expectations, and cultural context (’set’) combined with the physical and social environment (’setting’) significantly shape psychedelic effects.^104^ Proper management of set and setting enhances therapeutic efficacy and positive subjective experiences, including mystical-type experiences linked to long-term mental health benefits.^105^ Conversely, neglecting these factors can lead to adverse reactions, highlighting the importance of careful management for patient safety.^106^

### 1.3 Interactions of Psychedelics with Ovarian Hormones

Several molecular targets implicated in the effects of psychedelics—such as 5-HT2A receptors, BDNF, and mTOR—are also modulated by sex hormones, including E2 and progesterone, indicating potential points of convergence between hormonal and psychedelic signaling pathways.

Animal studies have identified sex-based differences in psychedelic response across behavioral and neurobiological outcomes. Female rodents consistently show greater head-twitch responses (HTR) to psychedelics like LSD and psilocybin than males, often linked to estrogen modulation.^107,108^ Estrogen replacement restores HTR after ovariectomy, suggesting hormone-dependent sensitivity. Additional studies show females also differ in locomotor activity, sensorimotor gating, and affective behaviors following psychedelic administration, with responses varying across the estrous cycle.^109–111^

Studies that have controlled for hormonal state have found that in preclinical investigations, rats in the proestrus and estrus phases—when estrogen levels are highest—showed significantly different responses to psychedelics (LSD and psilocin) in terms of locomotor activity and sensorimotor gating, respectively, compared to those in metestrus and diestrus.^7^ These effects have been linked to fluctuations in ovarian hormone levels, with estrogen and progesterone modulating 5-HT2A receptor expression and downstream signaling cascades.

Psychedelics engage neuroplasticity-related pathways, including BDNF and mTOR, which are also regulated by ovarian hormones. E2 enhances BDNF expression in the hippocampus and prefrontal cortex—key regions in psychedelic action—supporting hormone-mediated modulation of synaptic plasticity.^30,45^ Estrogen’s antidepressant effects are partly mediated through BDNF, with sex-specific sensitivity observed in animal models. For example, female rats show greater antidepressant-like responses to ketamine—an NMDA receptor antagonist also known to upregulate BDNF expression—compared to males; this effect is lost after ovariectomy and restored with estrogen and progesterone.^112^ While ketamine is not a classic psychedelic, this finding highlights the influence of ovarian hormones on BDNF-mediated plasticity, which may be relevant to understanding sex differences in response to psychedelic compounds with similar downstream effects.^113^

Preliminary clinical data suggest possible sex differences in psychedelic response. For example, Dudysová et al. (2020) reported differential subjective and emotional effects of LSD between men and women.^114^ However, most human studies have not yet included menstrual phase or hormonal contraceptive use as formal variables, limiting insight into the influence of hormonal state.

One possible factor contributing to these differences is the interaction between psychedelics and endocrine axes. Classic psychedelics can affect hypothalamic function and have been proposed to influence gonadotropin-releasing hormone (GnRH) secretion, with downstream effects on luteinizing hormone (LH) and follicle-stimulating hormone (FSH).^66^ Activation of 5-HT2A receptors in the hypothalamic paraventricular nucleus has been shown to mediate the release of neuroendocrine hormones, including ACTH, oxytocin, and prolactin, following serotonergic stimulation.^115^

In line with this, psychedelics have been shown to acutely increase circulating cortisol and prolactin levels.^9^ Elevated concentrations of these hormones are known to suppress reproductive hormone secretion and may contribute to menstrual irregularities when chronically dysregulated.^116–118^

The subjective dimension of psychedelic effects may also be shaped by hormonal state. Mystical-type experiences, which are associated with positive clinical outcomes,^119^ arise from a complex interplay of neurobiological, cognitive, and emotional processes, including large-scale brain network dynamics and affective modulation (see Section 1.2). Although no studies to date have demonstrated a direct influence of ovarian hormones on the intensity of mystical-type experiences, fluctuations in estrogen and progesterone are known to modulate emotion processing, limbic reactivity, and functional connectivity within brain networks engaged during psychedelic states, such as the default mode, salience, and executive control networks.^58,120^ These hormone-sensitive effects may influence the psychedelic experience and contribute to sex-specific variability in subjective response.

## 2 Discussion

The intersection of psychedelics and women’s hormonal cycles presents an untapped frontier in the field of psychopharmacology. The mechanisms reviewed in this paper suggest that MC phases and their accompanying hormonal changes may influence psychedelic mechanisms, impacting the subjective experience and psychological and other outcomes. Given the central role of ovarian hormones in modulating neurotransmitter systems affected by psychedelics, it is paramount that future research incorporates MC measurements to capture the nuances of these interactions fully.

Both psychedelics and ovarian hormones affect functioning of the serotonergic^17^ and other neurological systems.^18,22^ The physiological effects of estrogen-related changes in serotonin efficacy and receptor distribution^26^ could influence the overall activity of a psychedelic substance. Hormone-related cyclic modulation of brain connectivity^42,50^ and interspheric communication^52^ mirrors action seen in psychedelic mechanisms.^103^ Further, oscillations in female hormones during the menstrual and life cycles significantly impact mood, cognition and behavior,^121,122^ in turn influencing mindset, a significant factor in safe and effective psychedelic use.^123^ Subsequently, it could be reasonably considered that the hormonal landscape of a female using psychedelics may modulate their experience and outcomes.

Higher E2 levels—such as those observed midcycle around ovulation—may enhance the effects of serotonergic psychedelics by increasing serotonin availability and receptor binding;^24,25,27^ Acutely, this could result in stronger or more prolonged psychedelic responses during high-estrogen phases in females. In contrast, P4 exerts complex, context-dependent effects on the serotonin system, showing both facilitatory and modulatory influences.^24,26^ Its impact may vary between healthy individuals and those with clinical conditions, where opposite patterns are sometimes observed (e.g.,^124,125^ ). Moreover, as P4 is metabolized into neuroactive steroids that modulate GABAergic tone, and given that GABA’s psychological effects follow an inverted U-shaped curve,^126^ surpassing optimal levels may paradoxically lead to adverse or attenuated effects. Thus, the effect of P4 is less predictable. It can even get more complicated since we has not yet addressed the interaction between the stress-response system (HPA axis) and the reproductive axis (HPG)^66,68^ —an essential layer in understanding hormonal modulation of psychedelic experiences. The most direct and informative path forward lies in empirical research that systematically examines the influence of endogenous hormones on psychedelic responses in real time. Advancing such inquiry is precisely the central aim of this perspective paper.

A conservative and ethical approach may recommend initiating and evaluating psychedelic treatment in women during the follicular phase, up to ovulation—a period characterized by low progesterone levels and relatively more predictable effects of E2. This phase is also commonly associated with more positive affective states in most women, suggesting, in light of the importance of set and setting, a window of reduced psychological vulnerability and increased resilience. Simultaneously, it is crucial to advance empirical research that directly examines the effects of psychedelics in women across different phases of the menstrual cycle. Such studies should adopt within-subject designs, include both clinical and nonclinical populations, and integrate biological, psychological, and neuroimaging measures. Additionally, another conservative guiding principle is to refrain from generalizing findings between clinical and nonclinical groups. Each study must strive to generate disorder-specific and phase-specific data, as outcomes may differ significantly depending on diagnosis and menstrual timing.

Understanding psychedelics’ synergies with hormonal action and their engagement with regulatory systems like the HPA axis is crucial for reproductive health and may offer innovative interventions in women’s health. The potential of psychedelics to treat women-specific conditions, including premenstrual syndrome (PMS) and premenstrual dysphoric disorder (PMDD), menstrual and sexual dysfunctions, and fertility concerns, demands an intricate exploration of the interaction of these substances with the relevant hormonal and systemic pathways.Another key reason to include hormone variables in psychedelic studies is to account for possible systematic variability. As an anecdote, some clinical trial protocols introduce psychedelic sessions every 4 weeks, making them likely to occur at similar menstrual phases for any given individual, introducing variability in outcomes between females. This observation is not specific to the field of psychedelic research; hormonal phase variability influencing research results has also been observed in neuroimaging studies in psychopharmacology.^127^ While the focus of this paper has been on classic psychedelics like psilocybin, LSD, and DMT, it is important to note that substances like MDMA share serotonergic affinity profiles with many classic psychedelic drugs and have shown sex-related differences.^127^ Moreover, the effects of MDMA on ovarian hormones and the MC have been considered in clinical research.^128,129^

Future research must engage in qualitative and quantitative assessments of the MC.^130^ MC measurements should be included at every time point; most importantly and cost-effectively is to collect both subjective self-reports and objective measures to assess: (1) MC day; (2) First day of the current cycle; (3) First day of the next cycle or length of the MC; and (4) Whether or not the MC length is highly predictable and regular; if irregular, the participant should report an average length range (e.g., 25–35 days) and interpretations of menstrual phase should be taken with reservations. The suggested self-reported measurements will help with situating the participant in their menstrual phase and improving the predictions. Note however that self-reported MC information is not accurate enough when stand alone (see Gloe et al. (2023) for elaboration on this matter^131^). To increase precision and to account for relevant variance, the following biological measures should be collected: (1) E2 and P4 in saliva or/and in blood; and (2) FSH/LH tests or basal body temperature (BBT) monitoring to detect ovulation. Furthermore, the development and validation of wearable devices that effectively track fertility-related measures^132^ should also be considered. Wearable devices that continuously monitor BBT or other physiological markers may provide more accurate ovulation data (such as the Oura ring https://www.jmir.org/2024/1/e45139/). Such comprehensive data collection will enable a better understanding of the impact and interaction of psychedelics in females, allowing for the development of more personalized approaches to psychedelic therapy that account for individual hormonal profiles. To date, no studies have systematically examined the influence of hormonal contraceptive use on psychedelic responses in human females, highlighting a key gap in the literature.

We further recommend to count whether or not the participant experiences PMS (using validated retrospective questionnaires such as the Premenstrual Symptoms Screening Tool (PSST)^133^ in the beginning of the study they participate in, and/or prospective questionnaires such as the Daily Record of Severity of Problems (DRSP)^134^ or the McMaster Premenstrual and Mood Symptom Scale (MAC-PMSS)^135^ to validate the diagnosis^136^). Knowing whether they experience PMS/PMDD using the prospective measures is important since there are notable, and sometime opposite direction, differences in brain function or reported symptoms in response to ovarian hormones fluctuations (see Dubel el al., 2020 ^137^ and Epperson et al., 2002,^124^ for example), and also we want to make sure that the primary outcome (such as depressed mood^135^) of the study is not masked by with PMDD/PME.

To complement these clinical strategies, preclinical research should also adapt to the unique neuroendocrine profiles of female subjects. Animal studies investigating psychedelics should incorporate estrous cycle monitoring and consider phase-specific effects when analyzing behavioral and neurobiological outcomes. Where feasible, hormonal manipulations (e.g., ovariectomy with hormone replacement) can be used to isolate the effects of E2 and progesterone on psychedelic action. Such approaches are critical for improving translational validity and uncovering hormone-dependent mechanisms of action. Much of the mechanistic evidence on psychedelics is derived from studies conducted exclusively in male animals, reflecting a pervasive sex bias in preclinical research. This represents a significant limitation, as biological sex may influence psychedelic effects, highlighting the need for more inclusive, sex-balanced studies.

Beyond the MC, the application of psychedelics in the context of menopause also warrants further exploration, considering the significant endocrine transition that occurs during this phase and its impact on mental health and neurological mechanisms.^132^

Improved guidance for both clinical and nonclinical uses of psychedelics in relation to the MC and female reproductive system is necessary to ensure safe and effective use for women. The emergence of these substances into mainstream therapeutic practices must be informed by robust evidence that elucidates the complex interplay between women’s biology and psychedelic pharmacodynamics. For truly informed consent, women must be made aware of how hormonal fluctuations could uniquely influence their psychedelic experiences and physiology.

## 3 Conclusions

In conclusion, emerging evidence indicates that menstrual cycle–related changes may influence psychedelic outcomes. Given our understanding of the independent mechanisms and effects of ovarian hormones and psychedelics, there is a clear rationale to investigate how hormonal fluctuations across the cycle may modulate these psychedelic response.This could be associated with menstrual-related cyclic modulation of brain connectivity, interactions of ovarian hormones with the serotonergic and other systems, or interactions with stress and ovarian hormones through the HPA axis. Furthermore, the modulation of psychedelic responses may arise not only from hormone–brain interactions but also indirectly via menstrual cycle–related psychological fluctuations, which can influence the internal and external context—commonly referred to as set and setting. Future work should not only continue to dissect the mechanisms by which psychedelics exert their effects in relation to female hormones but also translate these findings into practice.
